# Crystal structure of a mono-bridged calix[4]arene

**DOI:** 10.1107/S2056989015010932

**Published:** 2015-06-13

**Authors:** Shimelis T. Hailu, Ray J. Butcher, Paul F. Hudrlik, Anne M Hudrlik

**Affiliations:** a Department of Chemistry, Howard University, 525 College Street NW, Washington, DC 20059, USA

**Keywords:** crystal structure, calix[4]arene, bridged calix[4]arene, flattened cone conformation, bromo­pent­oxy chain, hydrogen bonding

## Abstract

The title compound, 5^2^-[(5-bromo­pent­yl)­oxy]-1^2^,1^14^,3^5^,5^5^-tetra-*tert*-butyl-1^7^,1^8^,1^9^,1^10^-tetra­hydro-1^6^ *H*,1^16^ *H*-1(4,12)-dibenzo[*b*,*e*][1,7]dioxa­cyclo­dodecina-3,5(1,3)-dibenzena­cyclo­hexa­phan-3^2^-ol, C_54_H_73_BrO_4_, was synthesized from the reaction of *tert*-butyl­calix[4]arene with 1,5-di­bromo­pentane using K_2_CO_3_ in CH_3_CN. The structure consists of a calixarene unit with a five-carbon bridge connecting two proximal phenolic O atoms, and with a bromo­pent­oxy chain on one of the remaining phenolic O atoms. The calixarene unit was found to have a flattened cone conformation with no solvent (or other guest) mol­ecule observed in the cavity. Two of the opposite phenyl rings lean outwards with fold angles of 136.2 (1) and 133.0 (1)° between the rings and the plane of the bridging methyl­ene C atoms, while the other two opposite rings form fold angles of 83.27 (9) and 105.46 (9)°. There is considerable disorder in this mol­ecule. One of the *tert*-butyl groups is disordered over two conformations with occupancies of 0.527 (5) and 0.473 (5). The bromo­pent­oxy chain is disordered over three configurations with occupancies of 0.418, 0.332 and 0.250. The five-carbon bridge connecting two proximal phenolic O atoms is disordered over two conformations with occupancies of 0.537 (7) and 0.463 (7).

## Abstract 2

The structure of the title compound, consists of a *tert*-butyl­calix[4]arene with a five-carbon bridge connecting two proximal phenolic O atoms, and with a bromo­pent­oxy chain on one of the remaining phenolic O atoms.

## Chemical context   

Calixarenes are macrocyclic mol­ecules made up of phenol and methyl­ene units, and are useful as host mol­ecules and as building blocks for larger systems. (Ikeda & Shinkai, 1997 ▸; Gutsche, 2008 ▸). Calix[4]arenes exist in four well-defined conformations, and conformational inter­conversion (by rotation around the methyl­ene bridges) is inhibited when the phenolic oxygen atoms are alkyl­ated with sufficiently large groups (Ikeda & Shinkai, 1997 ▸). Calix[4]arenes in the cone conformation, which are tetra-*O*-alkyl­ated with bulky groups, generally adopt a flattened conformation (flattened or pinched cone, approximate *C*_2*v*_ symmetry) in the solid state; in solution they experience conformational mobility between flattened cones (Conner *et al.*, 1991 ▸; Arduini *et al.*, 1995 ▸, 1996*b* ▸; Drew *et al.*, 1997 ▸; Hudrlik *et al.*, 2007 ▸, 2013 ▸; Hailu *et al.*, 2012 ▸, 2013 ▸). Rigidified cone calixarenes (approximate *C*_4*v*_ symmetry) have been prepared by forming di­ethyl­ene glycol ether bridges between proximal phenolic oxygen atoms (Arduini *et al.*, 1995 ▸). In an effort to make a rigid cone calix[4]arene, we sought a strategy that would enable bridging of the phenolic oxygen atoms by the reactions of a calix[4]arene with 1,5-di­bromo­pentane. The reaction, using K_2_CO_3_ in CH_3_CN, gave a mixture consisting primarily of a bis-calixarene and a mono-bridged calixarene (Hudrlik *et al.*, 2013 ▸). In the present work, the X-ray crystal structure of the mono-bridged calixarene, the title compound, is described.
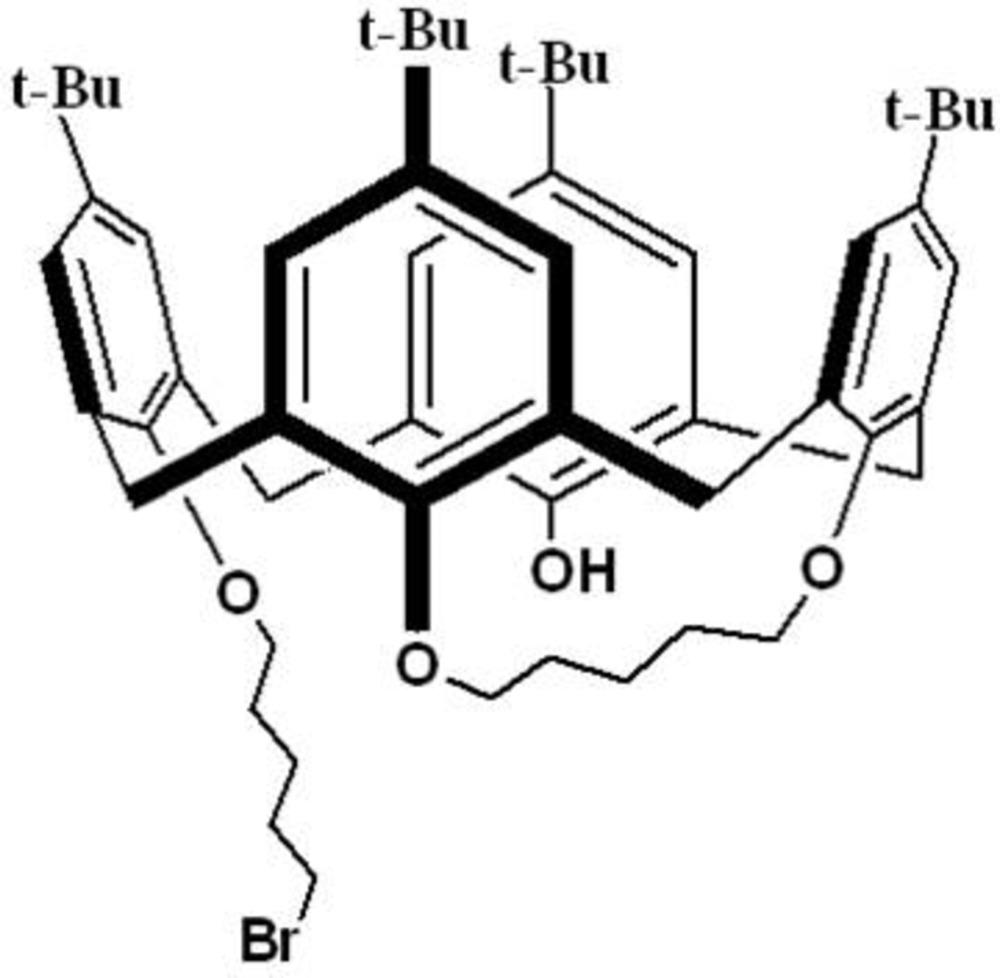


## Structural commentary   

**Figure 1:**
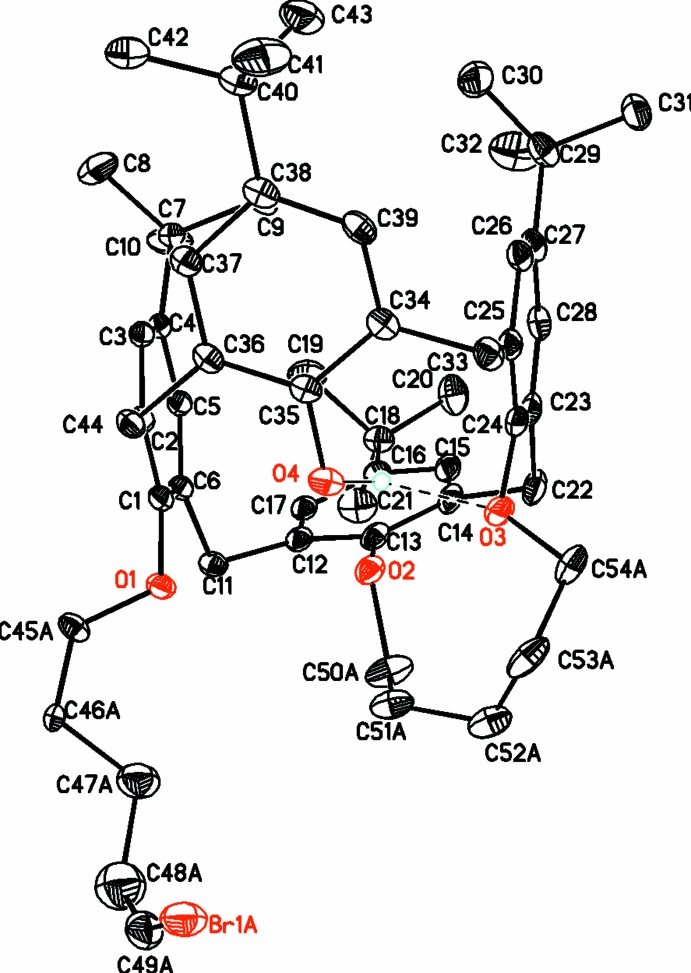
Diagram showing the atomic arrangement and atom-numbering scheme in the major component. Atomic displacement ellipsoids are drawn at the 30% level. H atoms are omitted for clarity.

The structure consists of a flattened-cone calix[4]arene having a five-carbon bridge joining two proximal phenolic oxygen atoms, and a bromo­pent­oxy chain attached to one of the remaining oxygen atoms. The mol­ecule (Fig. 1 ▸) has a relatively rigid framework with a semi-flexible bridge and a flexible side chain. The mol­ecule is inherently chiral, but crystallizes in a centrosymmetric space group; therefore both enanti­omers have to be present in the unit cell in equal amounts. However, the mol­ecule is disordered such that both enanti­omers involving the conformation adopted by the bridging atoms are present in the asymmetric unit. In one of the two enanti­omers, the bridging group links O3 and O2, and O3 and O4 in the other. The flexible side chain is disordered over three conformations. The diagrams show only the major component for the disordered regions.

The flattening of the calixarene cone could be observed by comparing distances between *para* carbon atoms of opposite phenolic rings. The distance between C4 and C27 is 5.698 (5) Å, while that between C16 and C38 is 9.390 (6) Å. The structure of a cone calix[4]arene is frequently described (Arduini *et al.*, 1996*b* ▸; Drew *et al.*, 1997 ▸) using the dihedral angles of the phenol rings with the plane of the bridging methyl­ene groups (C11, C22, C33, and C44). For the title compound, the aromatic rings attached to O2 and O4 are inclined outward, making fold angles of 136.2 (1) and 133.0 (1)°, respectively, while those attached to O1 and O3 are almost perpendicular to this plane, making dihedral angles of 83.27 (9) and 105.46 (9)°, respectively.

The fold angles reported here for the title compound are similar to those reported for other flattened cone calixarenes as referenced above. The joining of two proximal phenolic oxygen atoms by one five-carbon bridge does not appear to prevent flattening of the cone structure in the title compound. By contrast, a calix[4]arene having both sets of proximal phenolic oxygen atoms joined by five-atom bridges (di­ethyl­ene glycol derivatives) (and with a simple guest) had equivalent fold angles of about 115–118° (Arduini *et al.*, 1996*a* ▸).

In the mol­ecule there are several weak intra­molecular C—H⋯O inter­actions (Table 1 ▸). In addition, there is a weak intra­molecular C—H⋯Br inter­action. 

## Supra­molecular features   

**Figure 2:**
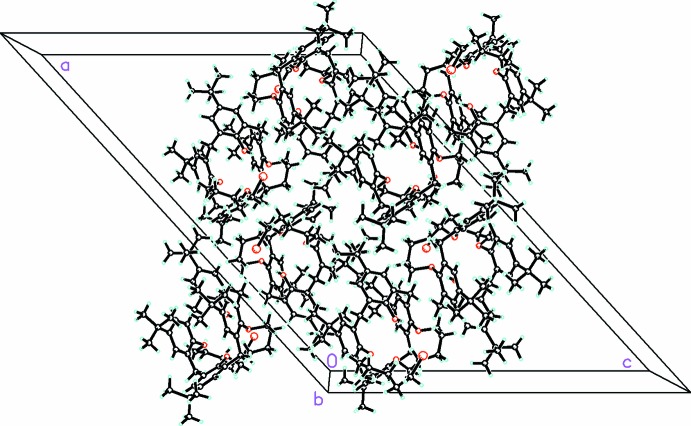
Packing diagram for the title compound, viewed along the *b* axis.

The bromine atoms in the disordered bromo­pent­oxy chain also participate in weak inter­molecular inter­actions, which link the mol­ecules into loosely associated dimers. Other than that, there are no close contacts between mol­ecules nor are there any significant inter­molecular or intra­molecular π–π inter­actions, possibly as a result of the conformation adopted by the calixarene skeleton due to the pentyl bridge between adjacent O atoms. A view of the packing is shown in Fig. 2 ▸.

## Database survey   

For the properties and conformational isomers of calix[4]arenes, see: Ikeda & Shinkai (1997 ▸); Gutsche (2008 ▸). For crystal structures of flattened-cone conformations of calix[4]arenes, see: Arduini *et al.* (1996*b* ▸); Drew *et al.* (1997 ▸); Hailu *et al.* (2012 ▸, 2013 ▸); Hudrlik *et al.* (2013 ▸). For other (solution) flattened-cone calix[4]arenes, see: Conner *et al.* (1991 ▸); Arduini *et al.* (1995 ▸); Hudrlik *et al.* (2007 ▸). For rigidified cone conformations of calix[4]arenes, see: Arduini *et al.* (1995 ▸); Arduini *et al.* (1996*a* ▸).

## Synthesis and crystallization   

The synthesis of the title compound was reported in the literature (Hudrlik *et al.*, 2013 ▸). Crystals for X-ray diffraction were obtained as follows. Approximately 10 mg of the white powdered solid compound was dissolved in a minimum amount of di­chloro­methane. The solution was filtered into a micro beaker and then methanol was added dropwise (final volume ratio about 4:1 methanol: di­chloro­methane). The beaker was covered loosely to allow slow evaporation of solvent. After a number of days, crystals suitable for X-ray analysis were obtained.

## Refinement   

Crystal data, data collection and structure refinement details are summarized in Table 2 ▸. There is considerable disorder in this mol­ecule. One of the *t*-butyl groups is disordered over two conformations with occupancies of 0.527 (5) and 0.473 (5) and each are constrained to the usual *tert*-butyl geometry. The bromo­pent­oxy chain is disordered over three conformations with occupancies of each conformer constrained to values of 0.418, 0.332 and 0.250 (total occupancy 1.000) which are similar to values of 0.417 (1), 0.331 (1) and 0.249 (1) obtained using the SAME command in *SHELXL2014* (Sheldrick, 2008 ▸). The five-carbon bridge connecting two proximal phenolic oxygen atoms is disordered over two conformations with occupancies of 0.537 (7) and 0.463 (7), such that one conformer links O2 and O3 while the other conformer links O3 and O4 and each conformer is constrained to have similar metric parameters as above. All hydrogen atoms attached to carbon atoms were refined using a riding model with idealized geometries (C—H = 0.95–0.98 Å with *U_iso_*(H) = 1.5*U*_eq_(C) for methyl H atoms and = 1.2*U*_eq_(C) for other H atoms).

## Supplementary Material

Crystal structure: contains datablock(s) I. DOI: 10.1107/S2056989015010932/hg5441sup1.cif

Structure factors: contains datablock(s) I. DOI: 10.1107/S2056989015010932/hg5441Isup2.hkl

CCDC reference: 1405207

Additional supporting information:  crystallographic information; 3D view; checkCIF report

## Figures and Tables

**Table 1:** Hydrogen-bond geometry (, )

*D*H*A*	*D*H	H*A*	*D* *A*	*D*H*A*
C48*A*H48*A*Br1*A* ^i^	0.99	2.95	3.612(11)	125
C48*B*H48*D*O1	0.99	2.57	3.227(16)	124
C51*A*H51*A*O4	0.99	2.66	3.588(10)	157
C51*B*H51*C*O2	0.99	2.65	3.597(10)	161
C51*B*H51*D*Br1*B*	0.99	2.99	3.939(8)	162

Symmetry code: (i) 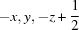.

**Table 2:** Experimental details

Crystal data	
Chemical formula	C_54_H_72_BrO_4_
*M*_r_	865.02
Crystal system, space group	Monoclinic, *C*2/*c*
Temperature (K)	123
*a*, *b*, *c* ()	34.730(5), 14.7386(7), 25.903(4)
()	132.36(2)
*V* (^3^)	9797(3)
*Z*	8
Radiation type	Cu *K*
(mm^1^)	1.46
Crystal size (mm)	0.46 0.33 0.10

Data collection	
Diffractometer	Agilent Xcalibur Ruby Gemini
Absorption correction	Analytical [*CrysAlis PRO* (Agilent, 2012 ▸), based on expressions derived by Clark Reid (1995 ▸)]
*T*_min_, *T*_max_	0.801, 0.948
No. of measured, independent and observed [*I* > 2(*I*)] reflections	20219, 9873, 6973
*R*_int_	0.030
(sin /)_max_ (^1^)	0.628

Refinement	
*R*[*F* ^2^ > 2(*F* ^2^)], *wR*(*F* ^2^), *S*	0.109, 0.337, 1.05
No. of reflections	9873
No. of parameters	649
No. of restraints	188
H-atom treatment	H-atom parameters constrained
_max_, _min_ (e ^3^)	1.27, 1.17

Computer programs: *CrysAlis PRO* (Agilent, 2012 ▸), *SIR92* (Altomare *et al.*, 1993 ▸), *SHELXL2014* (Sheldrick, 2015 ▸) and *SHELXTL* (Sheldrick, 2008 ▸).

## supplementary crystallographic information

### Crystal data

**Table:**

C_54_H_72_BrO_4_	*F*(000) = 3704
*M**_r_* = 865.02	*D*_x_ = 1.173 Mg m^−^^3^
Monoclinic, *C*2/*c*	Cu *K*α radiation, λ = 1.54184 Å
*a* = 34.730 (5) Å	Cell parameters from 5121 reflections
*b* = 14.7386 (7) Å	θ = 3.4–75.5°
*c* = 25.903 (4) Å	µ = 1.46 mm^−^^1^
β = 132.36 (2)°	*T* = 123 K
*V* = 9797 (3) Å^3^	Prism, colorless
*Z* = 8	0.46 × 0.33 × 0.10 mm

### Data collection

**Table:**

Agilent Xcalibur Ruby Gemini diffractometer	6973 reflections with *I* > 2σ(*I*)
Detector resolution: 10.5081 pixels mm^-1^	*R*_int_ = 0.030
ω scans	θ_max_ = 75.7°, θ_min_ = 3.4°
Absorption correction: analytical [*CrysAlis PRO* (Agilent, 2012), based on expressions derived by Clark & Reid (1995)]	*h* = −43→43
*T*_min_ = 0.801, *T*_max_ = 0.948	*k* = −18→13
20219 measured reflections	*l* = −28→32
9873 independent reflections	

### Refinement

**Table:**

Refinement on *F*^2^	188 restraints
Least-squares matrix: full	Hydrogen site location: inferred from neighbouring sites
*R*[*F*^2^ > 2σ(*F*^2^)] = 0.109	H-atom parameters constrained
*wR*(*F*^2^) = 0.337	*w* = 1/[σ^2^(*F*_o_^2^) + (0.1859*P*)^2^ + 21.3411*P*] where *P* = (*F*_o_^2^ + 2*F*_c_^2^)/3
*S* = 1.05	(Δ/σ)_max_ < 0.001
9873 reflections	Δρ_max_ = 1.27 e Å^−^^3^
649 parameters	Δρ_min_ = −1.17 e Å^−^^3^

### Special details

**Table:**

Experimental. Absorption correction: CrysAlisPro (Agilent Technologies, 2012) Analytical numeric absorption correction using a multifaceted crystal model based on expressions derived by R.C. Clark & J.S. Reid. (Clark, R. C. & Reid, J. S. (1995). Acta Cryst. A51, 887-897)
Geometry. All e.s.d.'s (except the e.s.d. in the dihedral angle between two l.s. planes) are estimated using the full covariance matrix. The cell e.s.d.'s are taken into account individually in the estimation of e.s.d.'s in distances, angles and torsion angles; correlations between e.s.d.'s in cell parameters are only used when they are defined by crystal symmetry. An approximate (isotropic) treatment of cell e.s.d.'s is used for estimating e.s.d.'s involving l.s. planes.

### Fractional atomic coordinates and isotropic or equivalent isotropic displacement parameters (Å^2^)

**Table:**

	*x*	*y*	*z*	*U*_iso_*/*U*_eq_	Occ. (<1)
O1	0.09438 (10)	0.75660 (17)	0.20459 (14)	0.0492 (6)	
O2	0.06851 (11)	0.5807 (2)	0.25782 (15)	0.0575 (7)	
O3	0.15164 (14)	0.5521 (3)	0.39836 (16)	0.0735 (10)	
O4	0.19201 (11)	0.67837 (19)	0.36417 (15)	0.0572 (7)	
C1	0.10989 (13)	0.6837 (2)	0.18839 (18)	0.0433 (7)	
C2	0.16175 (13)	0.6790 (2)	0.21800 (19)	0.0429 (7)	
C3	0.17639 (13)	0.6063 (2)	0.20057 (18)	0.0432 (7)	
H3A	0.2113	0.6035	0.2199	0.052*	
C4	0.14227 (13)	0.5372 (2)	0.15601 (17)	0.0420 (7)	
C5	0.09167 (13)	0.5429 (3)	0.12914 (18)	0.0433 (7)	
H5A	0.0676	0.4961	0.0990	0.052*	
C6	0.07494 (13)	0.6148 (2)	0.14485 (18)	0.0430 (7)	
C7	0.16228 (14)	0.4522 (3)	0.14695 (19)	0.0470 (8)	
C8	0.1990 (3)	0.4754 (4)	0.1355 (4)	0.0903 (19)	
H8A	0.2302	0.5054	0.1773	0.135*	
H8B	0.1813	0.5161	0.0953	0.135*	
H8C	0.2091	0.4196	0.1267	0.135*	
C9	0.1921 (3)	0.3975 (4)	0.2137 (3)	0.099 (2)	
H9A	0.1684	0.3798	0.2203	0.149*	
H9B	0.2205	0.4343	0.2534	0.149*	
H9C	0.2065	0.3429	0.2104	0.149*	
C10	0.11894 (19)	0.3945 (4)	0.0855 (3)	0.0880 (18)	
H10A	0.0955	0.3753	0.0920	0.132*	
H10B	0.1339	0.3409	0.0823	0.132*	
H10C	0.0994	0.4300	0.0423	0.132*	
C11	0.02081 (13)	0.6100 (3)	0.1203 (2)	0.0497 (8)	
H11A	0.0162	0.6605	0.1410	0.060*	
H11B	−0.0063	0.6154	0.0688	0.060*	
C12	0.01559 (13)	0.5207 (3)	0.1430 (2)	0.0495 (9)	
C13	0.04382 (14)	0.5066 (3)	0.2147 (2)	0.0538 (9)	
C14	0.04785 (15)	0.4207 (3)	0.2397 (2)	0.0594 (11)	
C15	0.02253 (16)	0.3493 (3)	0.1929 (2)	0.0597 (11)	
H15A	0.0257	0.2904	0.2104	0.072*	
C16	−0.00736 (15)	0.3597 (3)	0.1216 (2)	0.0541 (9)	
C17	−0.01004 (14)	0.4469 (3)	0.0979 (2)	0.0494 (8)	
H17A	−0.0301	0.4561	0.0495	0.059*	
C18	−0.03370 (18)	0.2789 (3)	0.0726 (3)	0.0674 (11)	
C19	−0.0115 (5)	0.2784 (8)	0.0351 (6)	0.093 (3)	0.527 (5)
H19A	−0.0203	0.3358	0.0101	0.140*	0.527 (5)
H19B	−0.0270	0.2279	0.0019	0.140*	0.527 (5)
H19C	0.0264	0.2712	0.0703	0.140*	0.527 (5)
C20	−0.0175 (5)	0.1931 (7)	0.1107 (6)	0.092 (3)	0.527 (5)
H20A	0.0204	0.1866	0.1420	0.138*	0.527 (5)
H20B	−0.0346	0.1426	0.0775	0.138*	0.527 (5)
H20C	−0.0275	0.1927	0.1382	0.138*	0.527 (5)
C21	−0.0899 (4)	0.2994 (8)	0.0187 (6)	0.096 (3)	0.527 (5)
H21A	−0.0948	0.3592	−0.0016	0.144*	0.527 (5)
H21B	−0.1049	0.2995	0.0400	0.144*	0.527 (5)
H21C	−0.1073	0.2532	−0.0181	0.144*	0.527 (5)
C19A	0.0037 (5)	0.2041 (9)	0.0900 (7)	0.093 (3)	0.473 (5)
H19D	0.0230	0.2236	0.0767	0.140*	0.473 (5)
H19E	−0.0160	0.1490	0.0641	0.140*	0.473 (5)
H19F	0.0282	0.1917	0.1402	0.140*	0.473 (5)
C20A	−0.0706 (5)	0.2282 (8)	0.0814 (7)	0.092 (3)	0.473 (5)
H20D	−0.0495	0.2103	0.1303	0.138*	0.473 (5)
H20E	−0.0863	0.1742	0.0516	0.138*	0.473 (5)
H20F	−0.0982	0.2697	0.0677	0.138*	0.473 (5)
C21A	−0.0718 (5)	0.2989 (8)	−0.0055 (6)	0.096 (3)	0.473 (5)
H21D	−0.0523	0.3168	−0.0189	0.144*	0.473 (5)
H21E	−0.0951	0.3483	−0.0163	0.144*	0.473 (5)
H21F	−0.0923	0.2444	−0.0316	0.144*	0.473 (5)
C22	0.08159 (18)	0.4024 (4)	0.3173 (2)	0.0717 (14)	
H22A	0.0754	0.4506	0.3376	0.086*	
H22B	0.0710	0.3439	0.3233	0.086*	
C23	0.13924 (16)	0.3989 (3)	0.3572 (2)	0.0579 (10)	
C24	0.17214 (16)	0.4717 (3)	0.3974 (2)	0.0545 (10)	
C25	0.22459 (16)	0.4690 (3)	0.43219 (18)	0.0496 (8)	
C26	0.24365 (16)	0.3908 (3)	0.42592 (19)	0.0497 (8)	
H26A	0.2796	0.3880	0.4499	0.060*	
C27	0.21240 (17)	0.3169 (3)	0.38625 (19)	0.0549 (9)	
C28	0.16030 (17)	0.3226 (3)	0.3527 (2)	0.0577 (10)	
H28A	0.1382	0.2725	0.3256	0.069*	
C29	0.23631 (19)	0.2323 (3)	0.3819 (2)	0.0678 (12)	
C30	0.2775 (3)	0.2597 (4)	0.3771 (4)	0.105 (2)	
H30A	0.2872	0.2061	0.3657	0.158*	
H30B	0.3085	0.2846	0.4221	0.158*	
H30C	0.2624	0.3056	0.3406	0.158*	
C31	0.2659 (3)	0.1788 (4)	0.4476 (3)	0.103 (2)	
H31A	0.2421	0.1590	0.4536	0.155*	
H31B	0.2935	0.2166	0.4874	0.155*	
H31C	0.2814	0.1255	0.4450	0.155*	
C32	0.1961 (3)	0.1803 (5)	0.3149 (4)	0.128 (3)	
H32A	0.1746	0.1440	0.3191	0.192*	
H32B	0.2137	0.1400	0.3063	0.192*	
H32C	0.1739	0.2229	0.2760	0.192*	
C33	0.26056 (17)	0.5490 (3)	0.4735 (2)	0.0560 (10)	
H33A	0.2932	0.5276	0.5193	0.067*	
H33B	0.2437	0.5925	0.4822	0.067*	
C34	0.27321 (15)	0.5967 (3)	0.43464 (19)	0.0494 (8)	
C35	0.23768 (13)	0.6570 (2)	0.38004 (19)	0.0451 (8)	
C36	0.24670 (13)	0.6931 (2)	0.33937 (19)	0.0459 (8)	
C37	0.29204 (14)	0.6694 (2)	0.3540 (2)	0.0489 (8)	
H37A	0.2982	0.6941	0.3263	0.059*	
C38	0.32866 (14)	0.6103 (3)	0.4086 (2)	0.0515 (9)	
C39	0.31812 (15)	0.5757 (3)	0.4480 (2)	0.0534 (9)	
H39A	0.3428	0.5360	0.4856	0.064*	
C40	0.37833 (16)	0.5821 (3)	0.4243 (2)	0.0599 (10)	
C41	0.4254 (2)	0.6240 (6)	0.4939 (3)	0.110 (2)	
H41A	0.4242	0.6091	0.5297	0.165*	
H41B	0.4246	0.6900	0.4889	0.165*	
H41C	0.4575	0.5997	0.5078	0.165*	
C42	0.3789 (2)	0.6145 (4)	0.3687 (3)	0.0833 (15)	
H42A	0.3748	0.6806	0.3640	0.125*	
H42B	0.3502	0.5860	0.3238	0.125*	
H42C	0.4121	0.5977	0.3826	0.125*	
C43	0.3818 (3)	0.4784 (4)	0.4270 (4)	0.099 (2)	
H43A	0.3842	0.4560	0.4647	0.149*	
H43B	0.4128	0.4596	0.4357	0.149*	
H43C	0.3506	0.4532	0.3823	0.149*	
C44	0.20332 (14)	0.7457 (2)	0.2734 (2)	0.0471 (8)	
H44A	0.2175	0.7792	0.2563	0.057*	
H44B	0.1879	0.7902	0.2837	0.057*	
Br1A	0.10640 (7)	1.07038 (13)	0.35595 (9)	0.0948 (4)	0.4179
C45A	0.0713 (8)	0.8223 (12)	0.1525 (9)	0.054 (2)	0.4179
H45A	0.0367	0.7999	0.1101	0.064*	0.4179
H45B	0.0930	0.8302	0.1408	0.064*	0.4179
C46A	0.0642 (5)	0.9145 (8)	0.1717 (6)	0.063 (2)	0.4179
H46A	0.0979	0.9473	0.2017	0.075*	0.4179
H46B	0.0388	0.9505	0.1286	0.075*	0.4179
C47A	0.0456 (4)	0.9071 (7)	0.2090 (5)	0.099 (3)	0.4179
H47A	0.0763	0.8982	0.2593	0.119*	0.4179
H47B	0.0239	0.8517	0.1921	0.119*	0.4179
C48A	0.0148 (4)	0.9845 (10)	0.2022 (6)	0.148 (6)	0.4179
H48A	−0.0165	0.9607	0.1915	0.177*	0.4179
H48B	0.0030	1.0232	0.1626	0.177*	0.4179
C49A	0.0461 (4)	1.0419 (8)	0.2682 (5)	0.088 (3)	0.4179
H49A	0.0230	1.0363	0.2778	0.106*	0.4179
H49B	0.0377	1.1026	0.2465	0.106*	0.4179
Br1B	0.06398 (9)	0.97616 (15)	0.34750 (11)	0.0948 (4)	0.3322
C45B	0.0774 (8)	0.8327 (15)	0.1554 (10)	0.054 (2)	0.3322
H45C	0.0469	0.8156	0.1066	0.064*	0.3322
H45D	0.1060	0.8535	0.1584	0.064*	0.3322
C46B	0.0633 (4)	0.9044 (10)	0.1826 (5)	0.063 (2)	0.3322
H46C	0.0383	0.9478	0.1446	0.075*	0.3322
H46D	0.0458	0.8747	0.1964	0.075*	0.3322
C47B	0.1095 (5)	0.9549 (9)	0.2434 (5)	0.099 (3)	0.3322
H47C	0.0987	1.0183	0.2405	0.119*	0.3322
H47D	0.1356	0.9566	0.2388	0.119*	0.3322
C48B	0.1357 (4)	0.9196 (13)	0.3143 (5)	0.148 (6)	0.3322
H48C	0.1735	0.9320	0.3462	0.177*	0.3322
H48D	0.1309	0.8530	0.3118	0.177*	0.3322
C49B	0.1144 (4)	0.9623 (13)	0.3443 (6)	0.127 (8)	0.3322
H49C	0.1193	1.0269	0.3394	0.153*	0.3322
H49D	0.1442	0.9491	0.3943	0.153*	0.3322
Br1C	0.11088 (12)	1.1110 (2)	0.37883 (16)	0.0948 (4)	0.2499
C45C	0.0792 (7)	0.8409 (19)	0.1686 (10)	0.054 (2)	0.2499
H45E	0.0572	0.8292	0.1181	0.064*	0.2499
H45F	0.1107	0.8739	0.1855	0.064*	0.2499
C46C	0.0492 (5)	0.9001 (12)	0.1795 (5)	0.063 (2)	0.2499
H46E	0.0318	0.9497	0.1446	0.075*	0.2499
H46F	0.0218	0.8631	0.1715	0.075*	0.2499
C47C	0.0833 (5)	0.9400 (12)	0.2506 (6)	0.099 (3)	0.2499
H47E	0.0839	1.0065	0.2461	0.119*	0.2499
H47F	0.1192	0.9176	0.2772	0.119*	0.2499
C48C	0.0693 (7)	0.9216 (9)	0.2929 (7)	0.148 (6)	0.2499
H48E	0.0848	0.8629	0.3174	0.177*	0.2499
H48F	0.0310	0.9163	0.2612	0.177*	0.2499
C49C	0.0884 (8)	0.9958 (7)	0.3464 (7)	0.088 (3)	0.2499
H49E	0.1172	0.9623	0.3892	0.106*	0.2499
H49F	0.0599	0.9933	0.3463	0.106*	0.2499
C50A	0.0401 (5)	0.6425 (10)	0.2710 (7)	0.094 (4)	0.463 (7)
H50A	0.0105	0.6718	0.2263	0.113*	0.463 (7)
H50B	0.0254	0.6040	0.2855	0.113*	0.463 (7)
C51A	0.0727 (4)	0.7159 (7)	0.3253 (5)	0.074 (3)	0.463 (7)
H51A	0.1045	0.7249	0.3330	0.088*	0.463 (7)
H51B	0.0527	0.7734	0.3066	0.088*	0.463 (7)
C52A	0.0886 (5)	0.6968 (9)	0.3942 (6)	0.090 (4)	0.463 (7)
H52A	0.0774	0.7488	0.4056	0.108*	0.463 (7)
H52B	0.0688	0.6430	0.3883	0.108*	0.463 (7)
C53A	0.1442 (5)	0.6802 (10)	0.4550 (6)	0.080 (5)	0.463 (7)
H53A	0.1516	0.6967	0.4980	0.096*	0.463 (7)
H53B	0.1653	0.7203	0.4517	0.096*	0.463 (7)
C54A	0.1609 (5)	0.5817 (8)	0.4611 (7)	0.067 (4)	0.463 (7)
H54A	0.1984	0.5755	0.5032	0.081*	0.463 (7)
H54B	0.1412	0.5417	0.4670	0.081*	0.463 (7)
C50B	0.1903 (4)	0.7516 (6)	0.3988 (6)	0.072 (3)	0.537 (7)
H50C	0.1915	0.8095	0.3806	0.087*	0.537 (7)
H50D	0.2218	0.7487	0.4492	0.087*	0.537 (7)
C51B	0.1430 (4)	0.7525 (6)	0.3906 (5)	0.071 (3)	0.537 (7)
H51C	0.1153	0.7158	0.3490	0.085*	0.537 (7)
H51D	0.1301	0.8156	0.3813	0.085*	0.537 (7)
C52B	0.1516 (6)	0.7168 (8)	0.4524 (8)	0.082 (4)	0.537 (7)
H52C	0.1447	0.7666	0.4709	0.098*	0.537 (7)
H52D	0.1887	0.6996	0.4895	0.098*	0.537 (7)
C53B	0.1183 (4)	0.6360 (6)	0.4372 (5)	0.067 (3)	0.537 (7)
H53C	0.1112	0.6383	0.4683	0.080*	0.537 (7)
H53D	0.0845	0.6400	0.3884	0.080*	0.537 (7)
C54B	0.1439 (4)	0.5463 (7)	0.4473 (5)	0.054 (2)	0.537 (7)
H54C	0.1775	0.5395	0.4960	0.065*	0.537 (7)
H54D	0.1209	0.4947	0.4357	0.065*	0.537 (7)

### Atomic displacement parameters (Å^2^)

**Table:**

	*U*^11^	*U*^22^	*U*^33^	*U*^12^	*U*^13^	*U*^23^
O1	0.0442 (13)	0.0459 (13)	0.0536 (14)	0.0097 (11)	0.0313 (12)	0.0066 (11)
O2	0.0475 (14)	0.0757 (19)	0.0552 (15)	0.0000 (13)	0.0370 (13)	0.0013 (14)
O3	0.089 (2)	0.099 (2)	0.0540 (16)	0.0335 (19)	0.0573 (17)	0.0165 (16)
O4	0.0504 (14)	0.0546 (16)	0.0563 (15)	0.0092 (12)	0.0318 (13)	−0.0037 (12)
C1	0.0392 (16)	0.0428 (17)	0.0449 (17)	0.0076 (14)	0.0271 (15)	0.0098 (14)
C2	0.0387 (16)	0.0412 (17)	0.0474 (18)	0.0036 (13)	0.0284 (15)	0.0102 (14)
C3	0.0367 (15)	0.0484 (19)	0.0441 (17)	0.0044 (14)	0.0270 (14)	0.0095 (15)
C4	0.0402 (16)	0.0463 (18)	0.0399 (16)	0.0072 (14)	0.0272 (14)	0.0105 (14)
C5	0.0381 (16)	0.0483 (18)	0.0406 (16)	0.0038 (14)	0.0253 (14)	0.0050 (15)
C6	0.0362 (16)	0.0484 (18)	0.0397 (16)	0.0083 (14)	0.0236 (14)	0.0098 (14)
C7	0.0457 (18)	0.0503 (19)	0.0499 (19)	0.0083 (15)	0.0342 (16)	0.0056 (16)
C8	0.106 (4)	0.077 (3)	0.143 (6)	−0.014 (3)	0.106 (5)	−0.024 (4)
C9	0.154 (6)	0.080 (4)	0.087 (4)	0.063 (4)	0.090 (4)	0.037 (3)
C10	0.060 (3)	0.078 (3)	0.102 (4)	−0.001 (2)	0.045 (3)	−0.036 (3)
C11	0.0343 (16)	0.055 (2)	0.0488 (19)	0.0059 (15)	0.0236 (15)	0.0035 (16)
C12	0.0337 (15)	0.067 (2)	0.0501 (19)	0.0026 (16)	0.0291 (15)	0.0044 (17)
C13	0.0374 (17)	0.079 (3)	0.050 (2)	−0.0047 (18)	0.0316 (16)	−0.0036 (19)
C14	0.0419 (18)	0.090 (3)	0.057 (2)	−0.002 (2)	0.0377 (18)	0.014 (2)
C15	0.0471 (19)	0.075 (3)	0.069 (3)	0.001 (2)	0.044 (2)	0.015 (2)
C16	0.0462 (19)	0.064 (2)	0.062 (2)	0.0034 (17)	0.0404 (19)	0.0053 (19)
C17	0.0372 (16)	0.064 (2)	0.0477 (19)	0.0041 (16)	0.0289 (15)	0.0044 (17)
C18	0.061 (2)	0.066 (3)	0.074 (3)	0.005 (2)	0.045 (2)	0.000 (2)
C19	0.087 (5)	0.083 (5)	0.101 (6)	0.000 (4)	0.060 (5)	−0.022 (4)
C20	0.104 (6)	0.069 (5)	0.088 (5)	−0.031 (4)	0.059 (5)	−0.004 (4)
C21	0.091 (6)	0.077 (5)	0.104 (7)	−0.025 (5)	0.059 (5)	−0.036 (5)
C19A	0.087 (5)	0.083 (5)	0.101 (6)	0.000 (4)	0.060 (5)	−0.022 (4)
C20A	0.104 (6)	0.069 (5)	0.088 (5)	−0.031 (4)	0.059 (5)	−0.004 (4)
C21A	0.091 (6)	0.077 (5)	0.104 (7)	−0.025 (5)	0.059 (5)	−0.036 (5)
C22	0.062 (2)	0.108 (4)	0.061 (3)	−0.002 (3)	0.048 (2)	0.019 (3)
C23	0.057 (2)	0.084 (3)	0.0407 (18)	0.000 (2)	0.0358 (18)	0.0148 (19)
C24	0.063 (2)	0.072 (3)	0.0410 (18)	0.011 (2)	0.0396 (18)	0.0130 (18)
C25	0.062 (2)	0.053 (2)	0.0363 (16)	0.0036 (17)	0.0344 (17)	0.0053 (15)
C26	0.055 (2)	0.050 (2)	0.0378 (17)	0.0030 (16)	0.0286 (16)	0.0046 (15)
C27	0.062 (2)	0.053 (2)	0.0349 (17)	0.0052 (18)	0.0264 (17)	0.0068 (15)
C28	0.062 (2)	0.061 (2)	0.0402 (18)	−0.0037 (19)	0.0306 (18)	0.0098 (17)
C29	0.077 (3)	0.053 (2)	0.047 (2)	0.008 (2)	0.031 (2)	0.0017 (18)
C30	0.161 (6)	0.084 (4)	0.130 (5)	0.037 (4)	0.122 (6)	0.021 (4)
C31	0.151 (5)	0.096 (4)	0.095 (4)	0.056 (4)	0.096 (4)	0.044 (3)
C32	0.109 (5)	0.105 (5)	0.125 (6)	−0.008 (4)	0.061 (5)	−0.051 (5)
C33	0.063 (2)	0.056 (2)	0.0367 (17)	0.0066 (19)	0.0290 (17)	0.0010 (16)
C34	0.0466 (18)	0.0436 (19)	0.0416 (18)	−0.0026 (15)	0.0231 (15)	−0.0068 (15)
C35	0.0390 (16)	0.0395 (17)	0.0442 (18)	−0.0017 (14)	0.0229 (15)	−0.0060 (14)
C36	0.0401 (17)	0.0359 (17)	0.0445 (18)	−0.0022 (13)	0.0214 (15)	−0.0015 (14)
C37	0.0439 (18)	0.0408 (18)	0.052 (2)	−0.0029 (15)	0.0280 (16)	−0.0017 (15)
C38	0.0408 (18)	0.0409 (18)	0.052 (2)	−0.0031 (14)	0.0224 (16)	−0.0082 (16)
C39	0.0454 (19)	0.0411 (19)	0.0454 (19)	0.0010 (15)	0.0191 (16)	−0.0006 (15)
C40	0.044 (2)	0.058 (2)	0.058 (2)	0.0086 (17)	0.0266 (18)	0.0007 (19)
C41	0.045 (2)	0.155 (7)	0.089 (4)	0.002 (3)	0.029 (3)	−0.029 (4)
C42	0.060 (3)	0.093 (4)	0.092 (4)	0.015 (3)	0.049 (3)	0.004 (3)
C43	0.106 (5)	0.068 (3)	0.133 (6)	0.029 (3)	0.084 (4)	0.018 (3)
C44	0.0427 (17)	0.0382 (17)	0.054 (2)	0.0040 (14)	0.0303 (16)	0.0082 (15)
Br1A	0.0980 (8)	0.0980 (9)	0.0863 (8)	0.0286 (7)	0.0613 (7)	−0.0016 (6)
C45A	0.063 (4)	0.038 (4)	0.044 (4)	0.009 (3)	0.030 (3)	0.000 (3)
C46A	0.097 (5)	0.060 (4)	0.076 (4)	0.030 (4)	0.077 (4)	0.031 (3)
C47A	0.112 (6)	0.098 (5)	0.087 (5)	0.027 (4)	0.066 (4)	0.000 (4)
C48A	0.152 (7)	0.147 (8)	0.148 (7)	0.008 (5)	0.103 (6)	−0.001 (5)
C49A	0.075 (5)	0.089 (6)	0.105 (6)	0.016 (4)	0.062 (5)	0.027 (5)
Br1B	0.0980 (8)	0.0980 (9)	0.0863 (8)	0.0286 (7)	0.0613 (7)	−0.0016 (6)
C45B	0.063 (4)	0.038 (4)	0.044 (4)	0.009 (3)	0.030 (3)	0.000 (3)
C46B	0.097 (5)	0.060 (4)	0.076 (4)	0.030 (4)	0.077 (4)	0.031 (3)
C47B	0.112 (6)	0.098 (5)	0.087 (5)	0.027 (4)	0.066 (4)	0.000 (4)
C48B	0.152 (7)	0.147 (8)	0.148 (7)	0.008 (5)	0.103 (6)	−0.001 (5)
C49B	0.121 (11)	0.163 (13)	0.121 (11)	0.036 (8)	0.091 (9)	−0.006 (8)
Br1C	0.0980 (8)	0.0980 (9)	0.0863 (8)	0.0286 (7)	0.0613 (7)	−0.0016 (6)
C45C	0.063 (4)	0.038 (4)	0.044 (4)	0.009 (3)	0.030 (3)	0.000 (3)
C46C	0.097 (5)	0.060 (4)	0.076 (4)	0.030 (4)	0.077 (4)	0.031 (3)
C47C	0.112 (6)	0.098 (5)	0.087 (5)	0.027 (4)	0.066 (4)	0.000 (4)
C48C	0.152 (7)	0.147 (8)	0.148 (7)	0.008 (5)	0.103 (6)	−0.001 (5)
C49C	0.075 (5)	0.089 (6)	0.105 (6)	0.016 (4)	0.062 (5)	0.027 (5)
C50A	0.069 (5)	0.118 (8)	0.110 (7)	−0.016 (5)	0.066 (5)	−0.043 (6)
C51A	0.064 (6)	0.071 (6)	0.094 (8)	−0.011 (5)	0.057 (6)	−0.031 (6)
C52A	0.099 (9)	0.095 (9)	0.103 (10)	0.002 (8)	0.079 (9)	−0.023 (8)
C53A	0.089 (9)	0.105 (13)	0.086 (8)	−0.052 (10)	0.075 (8)	−0.057 (9)
C54A	0.086 (8)	0.081 (9)	0.064 (7)	−0.029 (6)	0.062 (7)	−0.024 (6)
C50B	0.069 (5)	0.055 (4)	0.096 (6)	−0.008 (4)	0.057 (4)	−0.018 (4)
C51B	0.081 (6)	0.047 (4)	0.107 (7)	0.009 (4)	0.072 (6)	0.001 (4)
C52B	0.081 (7)	0.081 (9)	0.104 (9)	−0.024 (7)	0.070 (7)	−0.041 (7)
C53B	0.067 (5)	0.085 (7)	0.068 (5)	−0.005 (5)	0.054 (5)	−0.022 (5)
C54B	0.069 (6)	0.065 (6)	0.042 (4)	−0.001 (4)	0.043 (4)	−0.007 (4)

### Geometric parameters (Å, º)

**Table:**

O1—C1	1.388 (4)	C33—H33A	0.9900
O1—C45A	1.39 (2)	C33—H33B	0.9900
O1—C45C	1.42 (3)	C34—C39	1.386 (6)
O1—C45B	1.49 (3)	C34—C35	1.401 (5)
O2—C13	1.372 (5)	C35—C36	1.391 (6)
O2—C50A	1.543 (11)	C36—C37	1.393 (5)
O3—C24	1.391 (5)	C36—C44	1.523 (5)
O3—C54B	1.466 (9)	C37—C38	1.397 (5)
O3—C54A	1.494 (12)	C37—H37A	0.9500
O4—C35	1.380 (4)	C38—C39	1.391 (6)
O4—C50B	1.430 (9)	C38—C40	1.539 (6)
C1—C6	1.389 (5)	C39—H39A	0.9500
C1—C2	1.405 (5)	C40—C41	1.527 (7)
C2—C3	1.386 (5)	C40—C42	1.530 (7)
C2—C44	1.523 (5)	C40—C43	1.530 (7)
C3—C4	1.389 (5)	C41—H41A	0.9800
C3—H3A	0.9500	C41—H41B	0.9800
C4—C5	1.390 (5)	C41—H41C	0.9800
C4—C7	1.526 (5)	C42—H42A	0.9800
C4—C27	5.698 (5)	C42—H42B	0.9800
C5—C6	1.395 (5)	C42—H42C	0.9800
C5—H5A	0.9500	C43—H43A	0.9800
C6—C11	1.528 (5)	C43—H43B	0.9800
C7—C10	1.515 (6)	C43—H43C	0.9800
C7—C9	1.516 (6)	C44—H44A	0.9900
C7—C8	1.531 (6)	C44—H44B	0.9900
C8—H8A	0.9800	Br1A—C49A	1.821 (8)
C8—H8B	0.9800	C45A—C46A	1.523 (7)
C8—H8C	0.9800	C45A—H45A	0.9900
C9—H9A	0.9800	C45A—H45B	0.9900
C9—H9B	0.9800	C46A—C47A	1.484 (8)
C9—H9C	0.9800	C46A—H46A	0.9900
C10—H10A	0.9800	C46A—H46B	0.9900
C10—H10B	0.9800	C47A—C48A	1.490 (9)
C10—H10C	0.9800	C47A—H47A	0.9900
C11—C12	1.502 (6)	C47A—H47B	0.9900
C11—H11A	0.9900	C48A—C49A	1.522 (9)
C11—H11B	0.9900	C48A—H48A	0.9900
C12—C17	1.392 (6)	C48A—H48B	0.9900
C12—C13	1.413 (6)	C49A—H49A	0.9900
C13—C14	1.387 (7)	C49A—H49B	0.9900
C14—C15	1.383 (7)	Br1B—C49B	1.821 (8)
C14—C22	1.521 (6)	C45B—C46B	1.523 (7)
C15—C16	1.390 (6)	C45B—H45C	0.9900
C15—H15A	0.9500	C45B—H45D	0.9900
C16—C17	1.399 (6)	C46B—C47B	1.484 (8)
C16—C18	1.517 (7)	C46B—H46C	0.9900
C16—C38	9.390 (6)	C46B—H46D	0.9900
C17—H17A	0.9500	C47B—C48B	1.490 (9)
C18—C20	1.463 (10)	C47B—H47C	0.9900
C18—C21	1.476 (10)	C47B—H47D	0.9900
C18—C19A	1.522 (10)	C48B—C49B	1.522 (9)
C18—C21A	1.524 (11)	C48B—H48C	0.9900
C18—C19	1.595 (10)	C48B—H48D	0.9900
C18—C20A	1.627 (10)	C49B—H49C	0.9900
C19—H19A	0.9800	C49B—H49D	0.9900
C19—H19B	0.9800	Br1C—C49C	1.821 (8)
C19—H19C	0.9800	C45C—C46C	1.523 (7)
C20—H20A	0.9800	C45C—H45E	0.9900
C20—H20B	0.9800	C45C—H45F	0.9900
C20—H20C	0.9800	C46C—C47C	1.484 (8)
C21—H21A	0.9800	C46C—H46E	0.9900
C21—H21B	0.9800	C46C—H46F	0.9900
C21—H21C	0.9800	C47C—C48C	1.490 (9)
C19A—H19D	0.9800	C47C—H47E	0.9900
C19A—H19E	0.9800	C47C—H47F	0.9900
C19A—H19F	0.9800	C48C—C49C	1.522 (9)
C20A—H20D	0.9800	C48C—H48E	0.9900
C20A—H20E	0.9800	C48C—H48F	0.9900
C20A—H20F	0.9800	C49C—H49E	0.9900
C21A—H21D	0.9800	C49C—H49F	0.9900
C21A—H21E	0.9800	C50A—C51A	1.510 (12)
C21A—H21F	0.9800	C50A—H50A	0.9900
C22—C23	1.514 (6)	C50A—H50B	0.9900
C22—H22A	0.9900	C51A—C52A	1.497 (13)
C22—H22B	0.9900	C51A—H51A	0.9900
C23—C28	1.388 (7)	C51A—H51B	0.9900
C23—C24	1.393 (7)	C52A—C53A	1.472 (14)
C24—C25	1.386 (6)	C52A—H52A	0.9900
C25—C26	1.393 (6)	C52A—H52B	0.9900
C25—C33	1.515 (6)	C53A—C54A	1.531 (15)
C26—C27	1.385 (6)	C53A—H53A	0.9900
C26—H26A	0.9500	C53A—H53B	0.9900
C27—C28	1.385 (6)	C54A—H54A	0.9900
C27—C29	1.543 (6)	C54A—H54B	0.9900
C28—H28A	0.9500	C50B—C51B	1.508 (10)
C29—C31	1.490 (6)	C50B—H50C	0.9900
C29—C32	1.511 (7)	C50B—H50D	0.9900
C29—C30	1.567 (8)	C51B—C52B	1.512 (13)
C30—H30A	0.9800	C51B—H51C	0.9900
C30—H30B	0.9800	C51B—H51D	0.9900
C30—H30C	0.9800	C52B—C53B	1.515 (11)
C31—H31A	0.9800	C52B—H52C	0.9900
C31—H31B	0.9800	C52B—H52D	0.9900
C31—H31C	0.9800	C53B—C54B	1.514 (11)
C32—H32A	0.9800	C53B—H53C	0.9900
C32—H32B	0.9800	C53B—H53D	0.9900
C32—H32C	0.9800	C54B—H54C	0.9900
C33—C34	1.515 (6)	C54B—H54D	0.9900
			
C1—O1—C45A	109.5 (4)	C36—C37—C38	121.6 (4)
C1—O1—C45C	121.7 (6)	C36—C37—H37A	119.2
C1—O1—C45B	109.6 (6)	C38—C37—H37A	119.2
C13—O2—C50A	120.6 (5)	C39—C38—C37	117.4 (4)
C24—O3—C54B	111.1 (5)	C39—C38—C40	120.6 (4)
C24—O3—C54A	122.0 (5)	C37—C38—C40	122.0 (4)
C35—O4—C50B	120.7 (4)	C39—C38—C16	68.7 (2)
O1—C1—C6	120.4 (3)	C37—C38—C16	69.3 (2)
O1—C1—C2	119.2 (3)	C40—C38—C16	132.6 (2)
C6—C1—C2	120.3 (3)	C34—C39—C38	122.9 (4)
C3—C2—C1	118.5 (3)	C34—C39—H39A	118.5
C3—C2—C44	118.9 (3)	C38—C39—H39A	118.5
C1—C2—C44	122.3 (3)	C41—C40—C42	107.6 (5)
C2—C3—C4	122.9 (3)	C41—C40—C43	111.2 (5)
C2—C3—H3A	118.6	C42—C40—C43	107.7 (5)
C4—C3—H3A	118.6	C41—C40—C38	108.6 (4)
C3—C4—C5	117.0 (3)	C42—C40—C38	113.0 (4)
C3—C4—C7	120.5 (3)	C43—C40—C38	108.7 (4)
C5—C4—C7	121.7 (3)	C40—C41—H41A	109.5
C3—C4—C27	91.4 (2)	C40—C41—H41B	109.5
C5—C4—C27	93.6 (2)	H41A—C41—H41B	109.5
C7—C4—C27	77.01 (19)	C40—C41—H41C	109.5
C4—C5—C6	122.3 (3)	H41A—C41—H41C	109.5
C4—C5—H5A	118.8	H41B—C41—H41C	109.5
C6—C5—H5A	118.8	C40—C42—H42A	109.5
C1—C6—C5	119.0 (3)	C40—C42—H42B	109.5
C1—C6—C11	121.9 (3)	H42A—C42—H42B	109.5
C5—C6—C11	118.8 (3)	C40—C42—H42C	109.5
C10—C7—C9	109.4 (5)	H42A—C42—H42C	109.5
C10—C7—C4	112.9 (3)	H42B—C42—H42C	109.5
C9—C7—C4	107.0 (3)	C40—C43—H43A	109.5
C10—C7—C8	106.8 (4)	C40—C43—H43B	109.5
C9—C7—C8	108.8 (4)	H43A—C43—H43B	109.5
C4—C7—C8	111.8 (3)	C40—C43—H43C	109.5
C7—C8—H8A	109.5	H43A—C43—H43C	109.5
C7—C8—H8B	109.5	H43B—C43—H43C	109.5
H8A—C8—H8B	109.5	C36—C44—C2	108.8 (3)
C7—C8—H8C	109.5	C36—C44—H44A	109.9
H8A—C8—H8C	109.5	C2—C44—H44A	109.9
H8B—C8—H8C	109.5	C36—C44—H44B	109.9
C7—C9—H9A	109.5	C2—C44—H44B	109.9
C7—C9—H9B	109.5	H44A—C44—H44B	108.3
H9A—C9—H9B	109.5	O1—C45A—C46A	114.7 (8)
C7—C9—H9C	109.5	O1—C45A—H45A	108.6
H9A—C9—H9C	109.5	C46A—C45A—H45A	108.6
H9B—C9—H9C	109.5	O1—C45A—H45B	108.6
C7—C10—H10A	109.5	C46A—C45A—H45B	108.6
C7—C10—H10B	109.5	H45A—C45A—H45B	107.6
H10A—C10—H10B	109.5	C47A—C46A—C45A	112.6 (7)
C7—C10—H10C	109.5	C47A—C46A—H46A	109.1
H10A—C10—H10C	109.5	C45A—C46A—H46A	109.1
H10B—C10—H10C	109.5	C47A—C46A—H46B	109.1
C12—C11—C6	108.5 (3)	C45A—C46A—H46B	109.1
C12—C11—H11A	110.0	H46A—C46A—H46B	107.8
C6—C11—H11A	110.0	C46A—C47A—C48A	117.3 (8)
C12—C11—H11B	110.0	C46A—C47A—H47A	108.0
C6—C11—H11B	110.0	C48A—C47A—H47A	108.0
H11A—C11—H11B	108.4	C46A—C47A—H47B	108.0
C17—C12—C13	118.2 (4)	C48A—C47A—H47B	108.0
C17—C12—C11	122.3 (4)	H47A—C47A—H47B	107.2
C13—C12—C11	118.9 (4)	C47A—C48A—C49A	112.4 (7)
O2—C13—C14	122.3 (4)	C47A—C48A—H48A	109.1
O2—C13—C12	116.9 (4)	C49A—C48A—H48A	109.1
C14—C13—C12	120.7 (4)	C47A—C48A—H48B	109.1
C15—C14—C13	118.8 (4)	C49A—C48A—H48B	109.1
C15—C14—C22	119.5 (4)	H48A—C48A—H48B	107.9
C13—C14—C22	121.7 (5)	C48A—C49A—Br1A	151.7 (6)
C14—C15—C16	123.0 (4)	C48A—C49A—H49A	98.7
C14—C15—H15A	118.5	Br1A—C49A—H49A	98.7
C16—C15—H15A	118.5	C48A—C49A—H49B	98.7
C15—C16—C17	116.9 (4)	Br1A—C49A—H49B	98.7
C15—C16—C18	121.0 (4)	H49A—C49A—H49B	103.9
C17—C16—C18	122.1 (4)	O1—C45B—C46B	101.1 (15)
C15—C16—C38	64.8 (2)	O1—C45B—H45C	111.6
C17—C16—C38	70.5 (2)	C46B—C45B—H45C	111.6
C18—C16—C38	134.3 (2)	O1—C45B—H45D	111.6
C12—C17—C16	122.3 (4)	C46B—C45B—H45D	111.6
C12—C17—H17A	118.9	H45C—C45B—H45D	109.4
C16—C17—H17A	118.9	C47B—C46B—C45B	112.5 (7)
C20—C18—C21	118.5 (7)	C47B—C46B—H46C	109.1
C20—C18—C16	111.9 (6)	C45B—C46B—H46C	109.1
C21—C18—C16	107.3 (5)	C47B—C46B—H46D	109.1
C16—C18—C19A	114.0 (6)	C45B—C46B—H46D	109.1
C16—C18—C21A	116.9 (6)	H46C—C46B—H46D	107.8
C19A—C18—C21A	109.7 (8)	C46B—C47B—C48B	117.3 (8)
C20—C18—C19	106.5 (7)	C46B—C47B—H47C	108.0
C21—C18—C19	107.4 (7)	C48B—C47B—H47C	108.0
C16—C18—C19	104.3 (5)	C46B—C47B—H47D	108.0
C16—C18—C20A	109.3 (6)	C48B—C47B—H47D	108.0
C19A—C18—C20A	102.9 (7)	H47C—C47B—H47D	107.2
C21A—C18—C20A	102.4 (7)	C47B—C48B—C49B	112.4 (7)
C18—C19—H19A	109.5	C47B—C48B—H48C	109.1
C18—C19—H19B	109.5	C49B—C48B—H48C	109.1
H19A—C19—H19B	109.5	C47B—C48B—H48D	109.1
C18—C19—H19C	109.5	C49B—C48B—H48D	109.1
H19A—C19—H19C	109.5	H48C—C48B—H48D	107.9
H19B—C19—H19C	109.5	C48B—C49B—Br1B	151.9 (7)
C18—C20—H20A	109.5	C48B—C49B—H49C	98.6
C18—C20—H20B	109.5	Br1B—C49B—H49C	98.6
H20A—C20—H20B	109.5	C48B—C49B—H49D	98.6
C18—C20—H20C	109.5	Br1B—C49B—H49D	98.6
H20A—C20—H20C	109.5	H49C—C49B—H49D	103.8
H20B—C20—H20C	109.5	O1—C45C—C46C	112 (2)
C18—C21—H21A	109.5	O1—C45C—H45E	109.2
C18—C21—H21B	109.5	C46C—C45C—H45E	109.2
H21A—C21—H21B	109.5	O1—C45C—H45F	109.2
C18—C21—H21C	109.5	C46C—C45C—H45F	109.2
H21A—C21—H21C	109.5	H45E—C45C—H45F	107.9
H21B—C21—H21C	109.5	C47C—C46C—C45C	112.6 (7)
C18—C19A—H19D	109.5	C47C—C46C—H46E	109.1
C18—C19A—H19E	109.5	C45C—C46C—H46E	109.1
H19D—C19A—H19E	109.5	C47C—C46C—H46F	109.1
C18—C19A—H19F	109.5	C45C—C46C—H46F	109.1
H19D—C19A—H19F	109.5	H46E—C46C—H46F	107.8
H19E—C19A—H19F	109.5	C46C—C47C—C48C	117.3 (8)
C18—C20A—H20D	109.5	C46C—C47C—H47E	108.0
C18—C20A—H20E	109.5	C48C—C47C—H47E	108.0
H20D—C20A—H20E	109.5	C46C—C47C—H47F	108.0
C18—C20A—H20F	109.5	C48C—C47C—H47F	108.0
H20D—C20A—H20F	109.5	H47E—C47C—H47F	107.2
H20E—C20A—H20F	109.5	C47C—C48C—C49C	112.4 (7)
C18—C21A—H21D	109.5	C47C—C48C—H48E	109.1
C18—C21A—H21E	109.5	C49C—C48C—H48E	109.1
H21D—C21A—H21E	109.5	C47C—C48C—H48F	109.1
C18—C21A—H21F	109.5	C49C—C48C—H48F	109.1
H21D—C21A—H21F	109.5	H48E—C48C—H48F	107.9
H21E—C21A—H21F	109.5	C48C—C49C—Br1C	151.4 (7)
C23—C22—C14	113.2 (3)	C48C—C49C—H49E	98.8
C23—C22—H22A	108.9	Br1C—C49C—H49E	98.8
C14—C22—H22A	108.9	C48C—C49C—H49F	98.8
C23—C22—H22B	108.9	Br1C—C49C—H49F	98.8
C14—C22—H22B	108.9	H49E—C49C—H49F	103.9
H22A—C22—H22B	107.8	C51A—C50A—O2	116.0 (8)
C28—C23—C24	118.3 (4)	C51A—C50A—H50A	108.3
C28—C23—C22	120.0 (4)	O2—C50A—H50A	108.3
C24—C23—C22	121.6 (5)	C51A—C50A—H50B	108.3
C25—C24—O3	118.5 (4)	O2—C50A—H50B	108.3
C25—C24—C23	121.4 (4)	H50A—C50A—H50B	107.4
O3—C24—C23	119.9 (4)	C52A—C51A—C50A	114.7 (10)
C24—C25—C26	117.9 (4)	C52A—C51A—H51A	108.6
C24—C25—C33	122.3 (4)	C50A—C51A—H51A	108.6
C26—C25—C33	119.8 (4)	C52A—C51A—H51B	108.6
C27—C26—C25	122.8 (4)	C50A—C51A—H51B	108.6
C27—C26—H26A	118.6	H51A—C51A—H51B	107.6
C25—C26—H26A	118.6	C53A—C52A—C51A	118.1 (10)
C28—C27—C26	117.2 (4)	C53A—C52A—H52A	107.8
C28—C27—C29	122.9 (4)	C51A—C52A—H52A	107.8
C26—C27—C29	119.9 (4)	C53A—C52A—H52B	107.8
C28—C27—C4	81.5 (2)	C51A—C52A—H52B	107.8
C26—C27—C4	84.1 (2)	H52A—C52A—H52B	107.1
C29—C27—C4	104.8 (2)	C52A—C53A—C54A	113.6 (10)
C27—C28—C23	122.4 (4)	C52A—C53A—H53A	108.8
C27—C28—H28A	118.8	C54A—C53A—H53A	108.8
C23—C28—H28A	118.8	C52A—C53A—H53B	108.8
C31—C29—C32	115.8 (6)	C54A—C53A—H53B	108.8
C31—C29—C27	109.5 (4)	H53A—C53A—H53B	107.7
C32—C29—C27	111.6 (4)	O3—C54A—C53A	112.1 (9)
C31—C29—C30	105.3 (5)	O3—C54A—H54A	109.2
C32—C29—C30	103.1 (5)	C53A—C54A—H54A	109.2
C27—C29—C30	111.2 (4)	O3—C54A—H54B	109.2
C29—C30—H30A	109.5	C53A—C54A—H54B	109.2
C29—C30—H30B	109.5	H54A—C54A—H54B	107.9
H30A—C30—H30B	109.5	O4—C50B—C51B	114.8 (7)
C29—C30—H30C	109.5	O4—C50B—H50C	108.6
H30A—C30—H30C	109.5	C51B—C50B—H50C	108.6
H30B—C30—H30C	109.5	O4—C50B—H50D	108.6
C29—C31—H31A	109.5	C51B—C50B—H50D	108.6
C29—C31—H31B	109.5	H50C—C50B—H50D	107.5
H31A—C31—H31B	109.5	C50B—C51B—C52B	115.4 (9)
C29—C31—H31C	109.5	C50B—C51B—H51C	108.4
H31A—C31—H31C	109.5	C52B—C51B—H51C	108.4
H31B—C31—H31C	109.5	C50B—C51B—H51D	108.4
C29—C32—H32A	109.5	C52B—C51B—H51D	108.4
C29—C32—H32B	109.5	H51C—C51B—H51D	107.5
H32A—C32—H32B	109.5	C51B—C52B—C53B	115.2 (10)
C29—C32—H32C	109.5	C51B—C52B—H52C	108.5
H32A—C32—H32C	109.5	C53B—C52B—H52C	108.5
H32B—C32—H32C	109.5	C51B—C52B—H52D	108.5
C25—C33—C34	111.2 (3)	C53B—C52B—H52D	108.5
C25—C33—H33A	109.4	H52C—C52B—H52D	107.5
C34—C33—H33A	109.4	C54B—C53B—C52B	112.7 (9)
C25—C33—H33B	109.4	C54B—C53B—H53C	109.0
C34—C33—H33B	109.4	C52B—C53B—H53C	109.0
H33A—C33—H33B	108.0	C54B—C53B—H53D	109.0
C39—C34—C35	118.1 (4)	C52B—C53B—H53D	109.0
C39—C34—C33	120.9 (4)	H53C—C53B—H53D	107.8
C35—C34—C33	120.8 (4)	O3—C54B—C53B	103.1 (6)
O4—C35—C36	119.2 (3)	O3—C54B—H54C	111.1
O4—C35—C34	119.9 (4)	C53B—C54B—H54C	111.1
C36—C35—C34	120.8 (3)	O3—C54B—H54D	111.1
C35—C36—C37	119.2 (3)	C53B—C54B—H54D	111.1
C35—C36—C44	119.1 (3)	H54C—C54B—H54D	109.1
C37—C36—C44	121.1 (4)		
			
C45A—O1—C1—C6	−85.4 (10)	O3—C24—C25—C26	−174.6 (3)
C45C—O1—C1—C6	−98.2 (11)	C23—C24—C25—C26	−0.3 (5)
C45B—O1—C1—C6	−94.2 (10)	O3—C24—C25—C33	2.7 (5)
C45A—O1—C1—C2	96.2 (10)	C23—C24—C25—C33	177.1 (3)
C45C—O1—C1—C2	83.4 (11)	C24—C25—C26—C27	0.6 (5)
C45B—O1—C1—C2	87.4 (10)	C33—C25—C26—C27	−176.9 (3)
O1—C1—C2—C3	−179.2 (3)	C25—C26—C27—C28	−0.8 (6)
C6—C1—C2—C3	2.4 (5)	C25—C26—C27—C29	−179.9 (4)
O1—C1—C2—C44	6.7 (5)	C25—C26—C27—C4	76.5 (3)
C6—C1—C2—C44	−171.7 (3)	C26—C27—C28—C23	0.8 (6)
C1—C2—C3—C4	−1.0 (5)	C29—C27—C28—C23	179.8 (4)
C44—C2—C3—C4	173.3 (3)	C4—C27—C28—C23	−78.1 (3)
C2—C3—C4—C5	−0.6 (5)	C24—C23—C28—C27	−0.5 (5)
C2—C3—C4—C7	−171.1 (3)	C22—C23—C28—C27	177.9 (3)
C2—C3—C4—C27	−95.4 (3)	C28—C27—C29—C31	−102.4 (5)
C3—C4—C5—C6	0.8 (5)	C26—C27—C29—C31	76.6 (6)
C7—C4—C5—C6	171.2 (3)	C4—C27—C29—C31	168.2 (4)
C27—C4—C5—C6	94.2 (3)	C28—C27—C29—C32	27.1 (7)
O1—C1—C6—C5	179.4 (3)	C26—C27—C29—C32	−153.9 (5)
C2—C1—C6—C5	−2.2 (5)	C4—C27—C29—C32	−62.3 (5)
O1—C1—C6—C11	−7.6 (5)	C28—C27—C29—C30	141.6 (5)
C2—C1—C6—C11	170.8 (3)	C26—C27—C29—C30	−39.4 (6)
C4—C5—C6—C1	0.6 (5)	C4—C27—C29—C30	52.2 (4)
C4—C5—C6—C11	−172.7 (3)	C24—C25—C33—C34	−105.1 (4)
C3—C4—C7—C10	−167.3 (4)	C26—C25—C33—C34	72.2 (4)
C5—C4—C7—C10	22.6 (5)	C25—C33—C34—C39	−95.1 (4)
C27—C4—C7—C10	108.8 (4)	C25—C33—C34—C35	79.2 (4)
C3—C4—C7—C9	72.3 (5)	C50B—O4—C35—C36	−94.8 (6)
C5—C4—C7—C9	−97.8 (5)	C50B—O4—C35—C34	87.9 (6)
C27—C4—C7—C9	−11.6 (4)	C39—C34—C35—O4	178.8 (3)
C3—C4—C7—C8	−46.8 (5)	C33—C34—C35—O4	4.3 (5)
C5—C4—C7—C8	143.2 (4)	C39—C34—C35—C36	1.5 (5)
C27—C4—C7—C8	−130.7 (4)	C33—C34—C35—C36	−172.9 (3)
C1—C6—C11—C12	−121.8 (4)	O4—C35—C36—C37	−178.1 (3)
C5—C6—C11—C12	51.2 (4)	C34—C35—C36—C37	−0.8 (5)
C6—C11—C12—C17	−99.2 (4)	O4—C35—C36—C44	−7.3 (5)
C6—C11—C12—C13	71.6 (4)	C34—C35—C36—C44	170.0 (3)
C50A—O2—C13—C14	−93.4 (8)	C35—C36—C37—C38	0.0 (5)
C50A—O2—C13—C12	89.5 (8)	C44—C36—C37—C38	−170.6 (3)
C17—C12—C13—O2	−179.8 (3)	C36—C37—C38—C39	0.0 (5)
C11—C12—C13—O2	9.2 (5)	C36—C37—C38—C40	178.7 (3)
C17—C12—C13—C14	3.0 (5)	C36—C37—C38—C16	50.6 (3)
C11—C12—C13—C14	−168.1 (3)	C35—C34—C39—C38	−1.6 (6)
O2—C13—C14—C15	−178.8 (3)	C33—C34—C39—C38	172.9 (4)
C12—C13—C14—C15	−1.7 (5)	C37—C38—C39—C34	0.8 (6)
O2—C13—C14—C22	−2.1 (6)	C40—C38—C39—C34	−177.9 (4)
C12—C13—C14—C22	175.0 (3)	C16—C38—C39—C34	−50.1 (3)
C13—C14—C15—C16	−0.7 (6)	C39—C38—C40—C41	−69.9 (6)
C22—C14—C15—C16	−177.5 (4)	C37—C38—C40—C41	111.4 (5)
C14—C15—C16—C17	1.7 (6)	C16—C38—C40—C41	−158.2 (4)
C14—C15—C16—C18	179.1 (4)	C39—C38—C40—C42	170.7 (4)
C14—C15—C16—C38	50.9 (3)	C37—C38—C40—C42	−8.0 (6)
C13—C12—C17—C16	−2.0 (5)	C16—C38—C40—C42	82.5 (5)
C11—C12—C17—C16	168.8 (3)	C39—C38—C40—C43	51.2 (6)
C15—C16—C17—C12	−0.3 (5)	C37—C38—C40—C43	−127.5 (5)
C18—C16—C17—C12	−177.6 (3)	C16—C38—C40—C43	−37.1 (5)
C38—C16—C17—C12	−46.9 (3)	C35—C36—C44—C2	−72.9 (4)
C15—C16—C18—C20	−7.9 (8)	C37—C36—C44—C2	97.7 (4)
C17—C16—C18—C20	169.4 (7)	C3—C2—C44—C36	−54.0 (4)
C38—C16—C18—C20	75.9 (7)	C1—C2—C44—C36	120.0 (4)
C15—C16—C18—C21	123.7 (7)	C1—O1—C45A—C46A	−166.2 (10)
C17—C16—C18—C21	−59.1 (7)	O1—C45A—C46A—C47A	−42.1 (18)
C38—C16—C18—C21	−152.6 (6)	C45A—C46A—C47A—C48A	−152.8 (11)
C15—C16—C18—C19A	−58.7 (8)	C46A—C47A—C48A—C49A	−105.6 (14)
C17—C16—C18—C19A	118.6 (8)	C47A—C48A—C49A—Br1A	5 (3)
C38—C16—C18—C19A	25.1 (8)	C1—O1—C45B—C46B	−179.1 (8)
C15—C16—C18—C21A	171.5 (7)	O1—C45B—C46B—C47B	82.1 (16)
C17—C16—C18—C21A	−11.3 (8)	C45B—C46B—C47B—C48B	−92.4 (17)
C38—C16—C18—C21A	−104.7 (7)	C46B—C47B—C48B—C49B	−91.6 (11)
C15—C16—C18—C19	−122.6 (6)	C47B—C48B—C49B—Br1B	73 (2)
C17—C16—C18—C19	54.6 (6)	C1—O1—C45C—C46C	163.2 (7)
C38—C16—C18—C19	−38.8 (6)	O1—C45C—C46C—C47C	73 (2)
C15—C16—C18—C20A	55.8 (7)	C45C—C46C—C47C—C48C	−124 (2)
C17—C16—C18—C20A	−126.9 (6)	C46C—C47C—C48C—C49C	−153.2 (13)
C38—C16—C18—C20A	139.6 (5)	C47C—C48C—C49C—Br1C	20 (4)
C15—C14—C22—C23	100.4 (5)	C13—O2—C50A—C51A	173.6 (9)
C13—C14—C22—C23	−76.2 (6)	O2—C50A—C51A—C52A	−103.6 (12)
C14—C22—C23—C28	−76.6 (6)	C50A—C51A—C52A—C53A	112.0 (13)
C14—C22—C23—C24	101.8 (5)	C51A—C52A—C53A—C54A	−84.5 (15)
C54B—O3—C24—C25	−103.6 (6)	C24—O3—C54A—C53A	168.3 (7)
C54A—O3—C24—C25	−76.3 (7)	C52A—C53A—C54A—O3	59.5 (13)
C54B—O3—C24—C23	82.0 (6)	C35—O4—C50B—C51B	−165.0 (6)
C54A—O3—C24—C23	109.2 (7)	O4—C50B—C51B—C52B	101.3 (11)
C28—C23—C24—C25	0.3 (5)	C50B—C51B—C52B—C53B	−120.5 (11)
C22—C23—C24—C25	−178.1 (3)	C51B—C52B—C53B—C54B	89.8 (12)
C28—C23—C24—O3	174.5 (3)	C24—O3—C54B—C53B	−176.2 (6)
C22—C23—C24—O3	−3.9 (5)	C52B—C53B—C54B—O3	−59.1 (10)

### Hydrogen-bond geometry (Å, º)

**Table:**

*D*—H···*A*	*D*—H	H···*A*	*D*···*A*	*D*—H···*A*
C48*A*—H48*A*···Br1*A*^i^	0.99	2.95	3.612 (11)	125
C48*B*—H48*D*···O1	0.99	2.57	3.227 (16)	124
C51*A*—H51*A*···O4	0.99	2.66	3.588 (10)	157
C51*B*—H51*C*···O2	0.99	2.65	3.597 (10)	161
C51*B*—H51*D*···Br1*B*	0.99	2.99	3.939 (8)	162

Symmetry code: (i) −*x*, *y*, −*z*+1/2.
